# Common Review Mission: reflections on a concurrent evaluation measure

**DOI:** 10.1186/1753-6561-6-S5-O20

**Published:** 2012-09-28

**Authors:** Pragati Singh, T Sundararaman, Rajani Ved, Padam Khanna

**Affiliations:** 1 National Health System Resource Centre, Delhi, India

## Introduction

One of the challenges that the National Rural Health Mission (NRHM) faces is its ability to evaluate the progress of the programme in diverse contexts and use such evidence to constantly improve programme design and implementation. The National Rural Health Mission has put in place several measures for concurrent evaluation to review the progress of the programme and inform management decisions. One such measure of evaluation is the Common Review Mission (CRM). The Common Review Mission meets once in a year. The paper assesses the effectiveness of Common Review Missions as a methodology for programme assessment, as a programme component leading to improved service delivery and as a tool for informing policy decisions.

## Methods

We did a document analysis of the common review mission reports available since 2007 to understand the composition of the mission, methods of assessment used and outputs. We examined progress on some key parameters as assessed and reported in these reports. We also consulted citations of reports of the mission in various research publications.

Key informant interviews were conducted with decision makers in public health system to understand the utility of Common Review Mission reports.

## Results and discussion

Since 2007, the Common Review Mission has met five times. All Common Review Missions are collaborative efforts of a multidisciplinary team of government functionaries, public health experts, civil society members and development partners to reflect and examine the changes achieved under the National Rural Health Mission. On an average, 170 such experts constituted the mission distributed in 12 to 15 teams. The administrative coordination is undertaken by the Ministry of Health. The National Health System Resource Centre leads the analysis, drafting, discussions and finalization of the report.

An analysis of five CRM reports shows that the most productive part of the evaluation process is the dialogue held with different states, which helps state officials to identify gaps and initiate corrective action. The structure and tools of information gathering used by the CRM teams have become increasingly refined over the years. These have become a template for much of subsequent monitoring visits by the National Rural Health Mission.

At the policy level, Common Review Mission is a major source of information for research publications on the programme - including the series on Indian health sector in Lancet, Indian Journal of Community Medicine and Journal of Global Health. The use of the reports in national policy level for planning and improving implementation has been rather limited. It is imperative that data from such concurrent evaluations measures are fed back into the process of planning and implementation of programmes.

## Competing interests

None declared

## Funding statement

None declared

